# Protective effect of fermented aloe extract on glutamate-induced cytotoxicity in HT22 cells

**DOI:** 10.1080/19768354.2022.2147584

**Published:** 2022-11-22

**Authors:** Ki Beom Jeon, Seong Hun Lee, Yong Seong Kwon, Jin Hong Beak, Hyeon Lee, Choong Je Ma

**Affiliations:** a R&DB Center, Beauty Science, Ltd., Sejong, Korea; b R&D center, KJM Aloe Co., Ltd., Seoul, Korea; c Department of Medical Biomaterials Engineering, College of Biomedical Science, Kangwon National University, Chuncheon, South Korea; d Institute of Bioscience and Biotechnology, Kangwon National University, Chuncheon, South Korea

**Keywords:** *Aloe arborescens*, aloenin, aloin, neuroprotection, oxidative stress

## Abstract

Excessive glutamate can cause oxidative stress in neuronal cells and this can significantly contribute to the etiology of neurodegenerative disease. The present study mainly aims to investigate that aloe extract (AE) and fermented aloe extract (FAE) could protect against glutamate-induced cytotoxicity by modulating oxidative stress. In this study, both AE and FAE showed potent neuroprotective activity by inhibiting ROS and Ca^2+^ concentration, increasing mitochondria membrane potential, and activating glutathione-related enzymes against glutamate-insulted neurotoxicity in HT22 cells. In addition, the neuroprotective activity of FAE was more potent than that of AE. HPLC analysis reveals that the chemical composition of FAE is different from that of AE. Especially, the contents of aloin A, aloin B and aloenin were higher in FAE than in AE. In conclusion, this study indicates that both AE and FAE may have effective neuroprotective activity in glutamate-insulted pathological conditions such as Alzheimer’s disease by managing oxidative stress.

## Introduction

Dementia is a neuro-disorder associated with various cognitive impairments in learning, computing, or language ability due to cerebral dysfunction (Crapper and Deboni, 1978). It is known that 50–60% of dementia cases are caused by Alzheimer's disease (AD), which exhibits the amyloid-*β* plaque induced by hyperphosphorylation of tau protein, neurofibrillary tangles, and eventual neuronal cell death due to hostile stress (Esch et al., 1990; Palop and Mucke, 2010; Sadigh-Eteghad et al., 2015). An etiology of AD has not yet been clearly established. Therefore, it is difficult to treat AD, and currently, there is no cure. In the early stage of dementia, when the symptoms are mild, it is hard to diagnose the disease, but as the disease progresses, neurons in various areas become damaged, which leads to disability in daily living. Drugs currently used for the treatment of dementia include donepezil, rivastagmine, and galantamine (Ballard, 2002; Melo et al., 2003). All of them are AChE inhibitors. However, these AChE inhibitors or N-methyl-D-aspartate (NMDA) receptor blockers are related to side effects such as dysfunction of the digestive system and frequent sleep. In these days, many clinical trials of medicine for Alzheimer's disease treatment have failed and it is urgent to develop a new medicine (Coyle et al., 1983; Weon et al., 2013).

*Aloe arborescens* has been widely used for traditional medicine to treat topical and oral diseases for centuries. It has the third largest distribution amongst the Aloe genus plants. It is a large succulent plant with many heads. It grows up to 2–3 meters and leaves are green and succulent with spikes. It is also widely distributed in the western Mediterranean, Australia, California, Japan, South Korea, and the Marshall Islands. In Korea, the juice of succulent leaves is used as a functional food material (Boudreau and Beland, 2006; Surjushe et al., 2008).

Many studies show that *Aloe arborescens* has various pharmacological activities such as fungicidal activity against *Trichophyton mentagropytes*, antimicrobial activity, antiviral activity, antitumor activity, antioxidant, anti-inflammatory activity, hepatoprotective activity, antidiabetic activity, wound healing and burn healing (Jones et al., 2002; Yao et al., 2009; Kumar et al., 2013; Li et al 2014; Chihara et al., 2015). It has also been reported that *Aloe arborescens* contains many bioactive constituents such as polysaccharides, flavonoids, anthraquinone, and pyrones (Viljoen et al., 2000; Licini et al., 2015; El Sayed et al., 2016)

Fermentation process is a kind of traditional method used to enhance the pharmacological activity of natural products. The fermentation process using lactic acid-related bacteria under low temperature and mild conditions is mainly used to increase activity of active substances without change of stability. Especially, *Lactobacillus* families can be usually used for these purposes because they conform to the regulation of Food and Drug Administration (FDA) as generally recognized as safe (GRAS) (Kim et al., 2014; Lee et al., 2016).

In this study, we checked whether the *Aloe arborescens* extract (AE) has neuroprotective activity and we also conducted a study on whether the fermented *Aloe arborescens* extract (FAE) improves neuroprotective activity. In addition, we evaluated various biomarkers such as ROS and Ca^2+^ concentration, mitochondria membrane potential, glutathione peroxidase, glutathione reductase and glutathione amount to confirm the mechanisms of pharmacological activity.

## Materials and methods

### Plant material

The dried *Aloe arborescens* (100 g) was extracted in 900 ml of distilled water at 100°C for 24 hours using the extractor installed with a vertical reflux condenser (GLHMR B1000, Global Lab, Siheung, Korea). The extracted samples were concentrated by rotary vacuum evaporator (Rotary Vacuum Evaporator N-N series, EYELA, Rikakikai Co., Tokyo, Japan) and lyophilized to freeze dryer (RV8, Edwards, Sweden). To obtain the fermented aloe extract, 100 g of dried *Aloe arboresecens* was extracted with 900 mL of distilled water at 100°C for 24 hours with a reflux condensed extractor (GLHMR B1000, Global Lab, Siheung, Korea). Then, the whole extracts were mixed with the following basal medium: 0.5% yeast extract, 1% peptone, 2% glucose, 0.01% magnesium sulfate, 0.005% manganese sulfate, 0.2% potassium phosphate, 0.1% polysorbate 80. After that, 3% (v/v) of *Lactobacillus brevis* were inoculated into a medium which was already autoclaved at 121°C for 30 minutes. The incubator was operated at 100 rpm and 37°C for 3 days. Then, the culture broth was lyophilized to make a powder by a freeze dryer (RV8, Edwards, Sweden). The powders were stored at −20°C before use.

### Cell culture

HT-22 cells were grown in Dulbecco’s modified Eagle’s medium (DMEM) added with 10% (v/v) fetal bovine serum (FBS) and 1% penicillin–streptomycin in an incubator with 5% CO_2_ ambient at 37°C. DMEM were obtained from Sigma-Aldrich (MO. USA) and FBS were obtained from Gibco-BRL (NY, USA).

### Cell viability test

HT22 cells were pretreated with different concentrations of AE and FAE (1, 10, or 100 μg/ml). After 1 hour, glutamate (4.0 mM) was treated in HT22 cells and 24 hours additional incubation was followed. The neuroprotective activity of AE and FAE was assessed by 3-(4,5-dimethylthiazol-2-yl)−2,5-diphenyltetrazolium bromide (MTT) assay. Cultured cells were incubated in 48-well plates at a density of 1.9 × 10^4^ cells/well. 150 μL of 1 mg/ml MTT solution was poured into every well. After 3 hours incubation at 37°C temperature and 5% CO_2_ conditions, the cell media were removed by suction. The formazan crystals were formed from viable cells and solved in 200 ml dimethylsulfoxide (DMSO). The optical density (OD) absorbance of the whole wells was measured at 540 nm using a microplate reader (BioTek EL808, BioTek Instruments, Winooski, VT, USA). Neuroprotective activity was determined by relative protection (%). Relative protection was calculated using the following equation:

### Measurement of cellular peroxide

The amount of cellular ROS was measured with the radical-sensitive [(OD of sample treated group–OD of glutamate treated group)/(OD of control treated group–OD of glutamate treated group)]×100(%), 2’,7’-dichlorofluorescein diacetate (DCF-DA) by using the method of Goodman and Mattson (Goodman and Mattson, 1996). HT22 cells were cultured onto 48-well plates. The different concentrations of AE or FAE were treated to cultured HT22 cells. After 1 hour incubation, an excessive amount of neurotoxic glutamate (4.0 mM) was administrated to the cells. After 8 h, cells were treated with 10 μM of DCF-DA for 1 hour. The cells were washed by phosphate-buffered saline (PBS) and extracted with 1% Triton X-100 after 30 minutes. Oxidized DCF fluorescence was detected by measuring the light emission of exciting cells at 528 and 485 nm of wavelength (BioTek EL808, BioTek Instruments, Winooski, VT, USA).

### Measurement of intracellular Ca^2+^

Intracellular Ca^2+^ was detected by Fura-2AM. The different concentrations of AE or FAE (1.0, 10.0 and 100.0 μg/ml) were treated to cultured HT22 cells. After 1 hour incubation, an excessive amount of neurotoxic glutamate (4.0 mM) was administrated to the cells. After 8 hours, cells were treated with 20 μM of Fura-2AM. The cells were washed with phosphate-buffered saline (PBS) and extracted with 1% Triton X-100 after 2 hours. Fluorescence was measured at 340 and 380 nm of excitation and 520 nm of emission (BioTek EL808, BioTek Instruments, Winooski, VT, USA).

### Measurement of mitochondrial membrane potential (ΔΨ_m_)

Mitochondrial membrane potential (ΔΨ_m_) level of HT22 cells was measured by rhodamine 123 (Rho 123). The different concentrations of AE or FAE (1.0, 10.0 and 100.0 μg/ml) were treated to cultured HT22 cells. After 1 hour incubation, an excessive amount of neurotoxic glutamate (4.0 mM) was administrated to the cells. After 24 hours, cells were treated with 10 μM of Rho123. The cells were washed by phosphate-buffered saline (PBS) and extracted with 1% Triton X-100 after 2 hours. Fluorescence was measured at 480 nm of excitation and 520 nm of emission (BioTek EL808, BioTek Instruments, Winooski, VT, USA).

### Measurement of glutathione content and antioxidant enzyme activity

HT22 cells were cultured in 6-well plates at a density of 3.4 × 10^4^ cells/well. The different concentrations of AE or FAE (1.0, 10.0 and 100.0 μg/ml) were treated to cultured HT22 cells. After 1 hour incubation, an excessive amount of neurotoxic glutamate (4.0 mM) was administrated to the cells. The culture cells were homogenized with 0.2 M phosphate buffer (pH 7.4) and centrifuged at 3000 g for 30 minutes at 4°C. The relative level of total glutathione (GSH) in the supernatant was measured by spectrophotometric way by using the enzymatic cycling method. To measure the total amount of GSH, a cell supernatant or GSH standard product was added to the reaction solution containing 5 units/ml GSSG-R and 0.3 mM NADPH. 0.5 mM DTNB was added to the reaction solution and reacted at 37°C for 30 s. Absorbance was measured at 412 nm for 2 minutes using a microplate reader (BioTek EL808, BioTek Instruments, Winooski, VT, USA). The amount of GSH was determined based on the standard value of the GSH standard.

Relative glutathione peroxidase (GPx) level was measured by the modified method of Brigelius-Flohé and Maiorino (2013). To measure the activity of GPx, the cell supernatants were added to phosphate buffer (pH7.2) containing 0.5 unit/ml glutathione reductase, 1 mM GSH and 0.2 mM NADPH was added, and finally 1.5 mM cumene hydroperoxide was added and reacted at 37°C for 3 minutes. The absorbance was measured at 340 nm for 2 minutes (BioTek EL808, BioTek Instruments, Winooski, VT, USA).

Glutathione reductase (GSSG-R) activity was measured by following the method of Carlberg and Mannervik (1985). To measure the activity of GSSG-R, the supernatant or GSSG-R control was added to phosphate buffer (pH 7.2) containing 1 mM oxidized glutathione (GSSG) and 0.1 mM NADPH. This solution reacted at 37°C for 2 minutes. Absorbance was detected at 340 nm for 2 minutes (BioTek EL808, BioTek Instruments, Winooski, VT, USA). The activity of GSSG-R was determined compared with the value of GSSG-R control.

### HPLC analysis of AE, FAE and active compounds

The HPLC analysis of three active compounds, aloin A, aloin B and aloenin, AE and FAE were performed on Dionex^TM^ Ultimate^TM^ 3000 HPLC system with LPG 3X00 pump, ACC-3000 auto sampler, column oven, and DAD-3000(RS) diode array UV/VIS detector. Separation was conducted using a Shiseido C18 column (4.6 mmI.D. × 250, 5 mm pore size) at 20°C. The mobile phase consisted of 0.1% trifluoroacetic acid (TFA) aqueous solution (A) and acetonitrile (B). The gradient program was optimized as follows: 25% B at 0–10 min, 25–40% B at 10–20 min, 40–55% B at 20–25 min, 55–80% B at 25–35 min and 80% at 35–40 min. The flow rate of mobile phase was at 1.0 ml/min and the injection volume was 20 μL. Compounds detected were set at 205 and 280 nm of UV wavelength.

### Statistical analysis

Data were presented as mean ± standard deviation (S.D.). Each experiment was replicated at least three times. All experimental results were statistically analyzed using one-way ANOVA and Tukey’s post hoc test with IBM SPSS Statistics software V26 (IBM, Armonk, NY, USA). Values of *p < 0.05, **p < 0.01, and ***p < 0.001 versus the glutamate-treated group and **^+^***p* < 0.05, **^++^***p* < 0.01, and **^+++^***p* < 0.001 versus the AE-treated group were showed statistically significant differences. All data were expressed as percentage compared with untreated control group which is set to 100% except for the relative neuroprotection of AE and FAE treated group.

## Results

Neuroprotection activity of aloe extract (AE) and fermented aloe extract (FAE) was evaluated by using glutamate insulted HT22 cells, a mouse hippocampal cells, as a model assay system. To investigate the neuroprotective activity of AE and FAE, AE and FAE were pretreated to the HT22 cells, and 1 hour after glutamate was administered to induce neurotoxicity. After 24 hours, cell viability was measured by MTT assay. In all of the experiments, trolox was used for the positive control and trolox was synthesized as a derivative of vitamin E and act as a water-soluble antioxidant (Scott et al., 1974).

**Figure 1.**
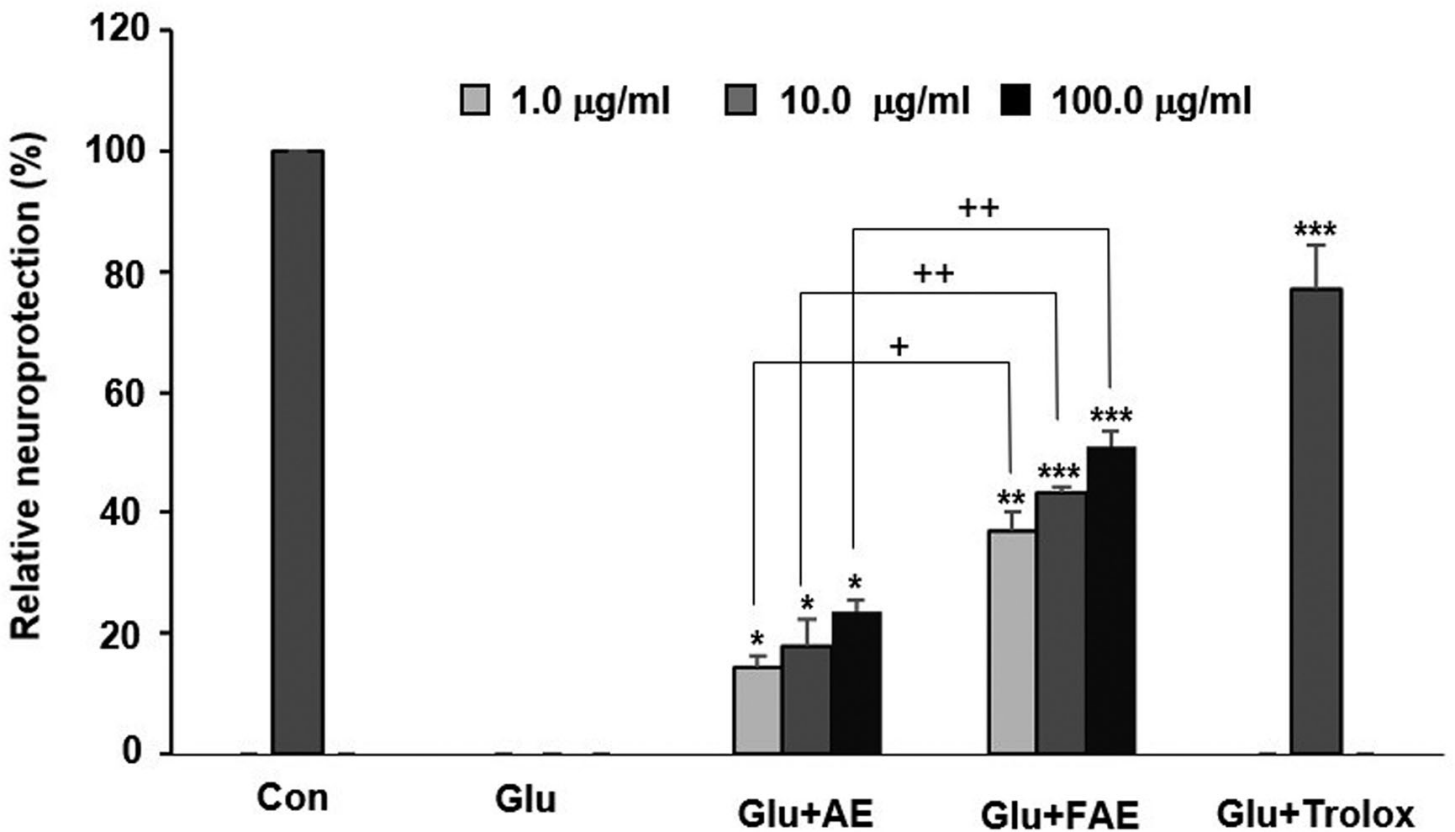
Effect of *A. arborescens* extract (AE: 1, 10 and 100 μg/ml) and fermented *A. arborescens* extract (FAE: 1, 10 and 100 μg/ml) on glutamate-induced death of HT22 cells. Data are means ± S.D. *p < 0.05, **p < 0.01, and ***p < 0.001 versus the glutamate-treated group and **^+^***p* < 0.05, **^++^***p* < 0.01, and **^+++^***p* < 0.001 versus the AE-treated group.

AE and FAE exhibited significant neuroprotective activity in a dose-dependent manner and most potent activity showed at the concentration of 100 μg/ml. However, the neuroprotective activity of FAE is more potent than that of AE. Cell viability of glutamate treated group was decreased from 100 to 56.8 ± 2.4% (data not shown). AE and FAE treated groups increased the relative protection to 23.7 ± 1.8%, 51.1 ± 2.4% at the concentration of 100 μg/ml, respectively. FAE treated group were more potent than AE treated group (Figure 1).

**Figure 2.**
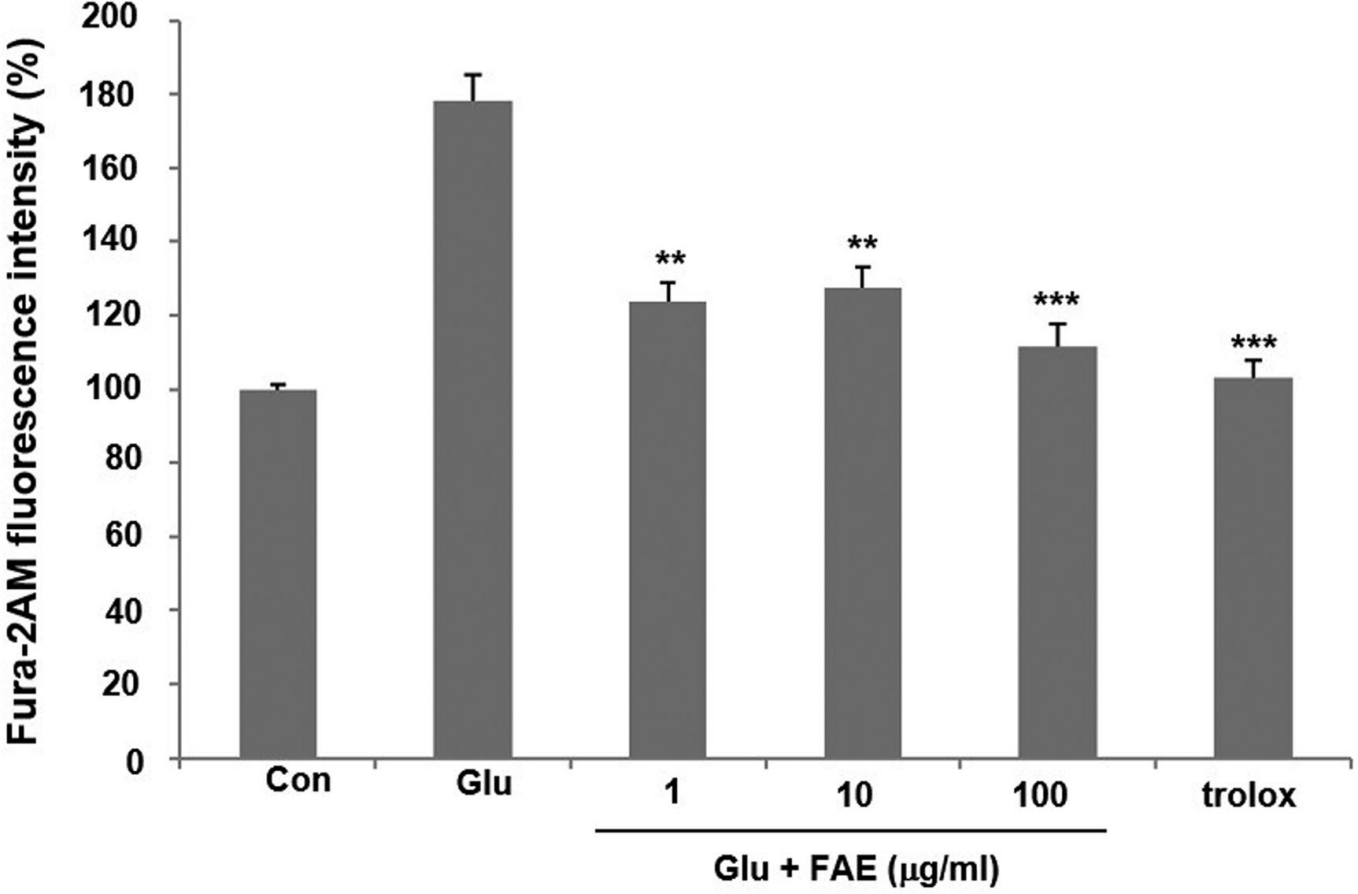
Effect of fermented *A. arborescens* extract (FAE: 1, 10 and 100 μg/ml) on intracellular Ca^2+^ concentration against glutamate induced neurotoxicity in HT22 cell. Data are means ± S.D. *p < 0.05, **p < 0.01, and ***p < 0.001 versus the glutamate-treated group

Mechanism of action of neuroprotective activity of FAE was evaluated. High concentrations of glutamate induce neuronal cell death through the effect of increasing the amount of cellular calcium ions in HT22 cells. In the glutamate-treated group, intracellular Ca^2+^ concentration increased by 178.3 ± 6.7% compared to the control group. However, 1, 10 and 100 μg/ml of FAE lowered the intracellular Ca^2+^ level increased by the treatment of glutamate by 123.8  ± 5.2, 127.6 ± 5.5, and 111.3 ± 6.2% of control one, respectively (Figure 2).

**Figure 3.**
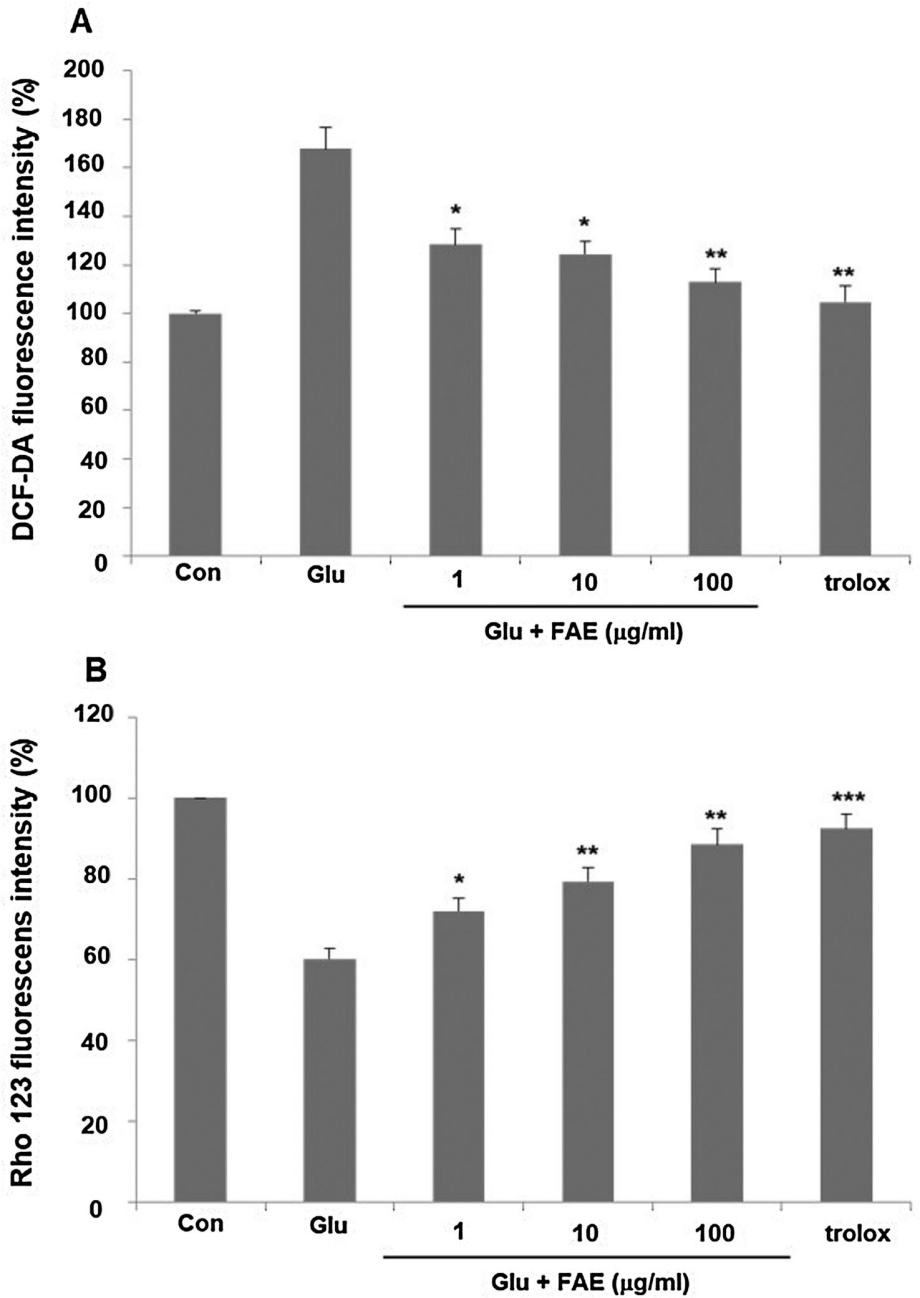
(A) Effect of fermented *A. arborescens* extract (FAE: 1, 10 and 100 μg/ml) on reactive oxygen species (ROS) production against glutamate induced neurotoxicity in HT22 cell. (B) Effect of fermented *A. arborescens* extract (FAE: 1, 10 and 100 μg/ml) on glutamate-induced disruption of mitochondrial membrane potential in HT22 cell. Data are means ± S.D. *p < 0.05, **p < 0.01, and ***p < 0.001 versus the glutamate-treated group.

Excess Ca^2+^ ions introduced into neuronal cells can regulate the activity of various enzymes such as phospholipases, calpain, and nNOS affected by Ca^2+^ and increase the production of free radicals or ROS as a result (Takata et al., 2020). We evaluated the effect of FAE on ROS production by using DCF-DA fluorescent dye. In the glutamate-treated group, intracellular ROS production was increased by 167.8 ± 8.9% compared to the control group. Pretreatment of 1 μg/ml, 10 μg/ml and 100 μg/ml of FAE decreased ROS level in the cells increased by the treatment of glutamate by 128.3 ± 6.4, 124.3 ± 5.2, and 112.9 ± 5.5%, respectively (Figure 3(A)).

Mitochondria are intracellular organelles that play an important role in the defense mechanisms of neuronal cell death against excitatory neurotoxicity caused by glutamate. The process of producing ATP in mitochondria is related to an electron transport system and an oxidative phosphorylation process. Mitochondria membrane potential is a major marker of mitochondrial function (Li et al., 2021) We evaluated the effect of FAE on mitochondria membrane potential by using rhodamine 123, a fluorescent dye. In the glutamate-treated group, mitochondria membrane potential was decreased by 60.2 ± 2.5% compared to the control group. Pretreatment of 1, 10 and 100 μg/ml of FAE recovered mitochondria membrane potential to 72.1 ± 3.2, 79.4 ± 3.3, and 88.5 ± 3.8% of control level, respectively (Figure 3(B)).

**Figure 4.**
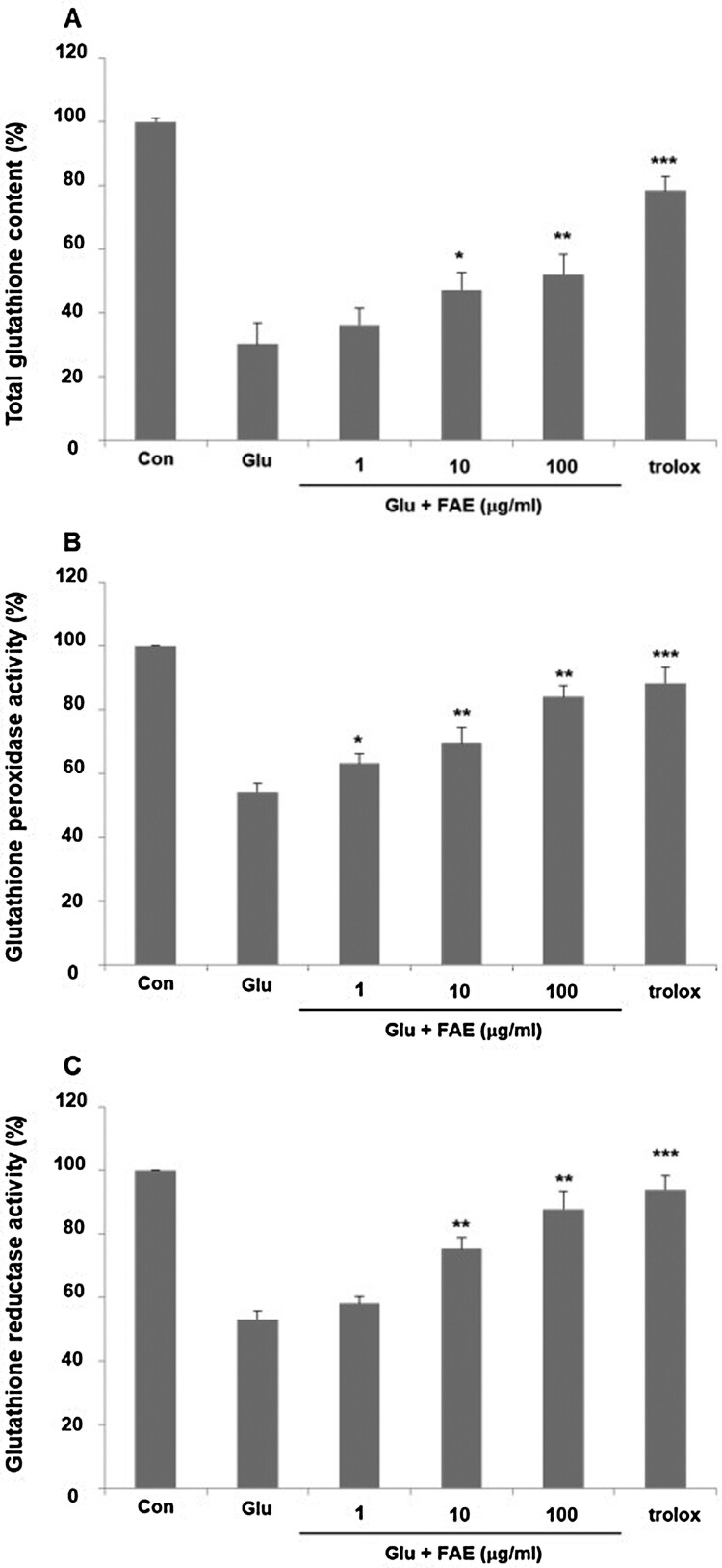
(A) Effect of fermented *A. arborescens* extract (FAE: 1, 10 and 100 μg/ml) on total glutathione amount against glutamate induced neurotoxicity in HT22 cell. (B) Effect of fermented *A. arborescens* extract (FAE: 1, 10 and 100 μg/ml) on glutathione peroxidase activity against glutamate induced neurotoxicity in HT22 cell. (C) Effect of fermented *A. arborescens* extract (FAE: 1, 10 and 100 μg/ml) on glutathione reductase activity against glutamate induced neurotoxicity in HT22 cell. Data are means ± S.D. *p < 0.05, **p < 0.01, and ***p < 0.001 versus the glutamate-treated group.

Cells develop various ways to deal with oxidative damage. Glutathione is an important molecule that removes nucleophilic toxicants such as free radicals and ROS. In normal cells, glutathione decreases oxidative radicals and returns to normal levels within a short time (Chen et al., 2021). However, glutamate treatment continuously decreases cellular glutathione and inactivate antioxidant enzymes such as superoxide dismutase, glutathione reductase and glutathione peroxidase (Fukui et al., 2010). As a result, free radicals such as O^2^^–^ and H_2_O_2_, which are toxic to cells, maintain high concentrations. Malfunction of glutathione metabolism causes oxidative damage, which leads to serious neurological diseases (Pallast et al., 2009). We evaluated the effect of FAE on glutathione amount. In the glutamate-treated group, total glutathione was decreased by 30.2 ± 1.8% compared to the control group. Pretreatment of 1, 10 and 100 μg/ml of FAE increased total glutathione amount decreased by the treatment of glutamate by 36.3 ± 2.4, 47.2 ± 3.2, and 52.1 ± 3.2%, respectively (Figure 4(A)). Furthermore, the effect of FAE on the activity of glutathione peroxidase and glutathione reductase was evaluated. In the glutamate-treated group, glutathione peroxidase was decreased by 54.3 ± 2.5% compared to the control group. Pretreatment of 1, 10 and 100 μg/ml of FAE increased activity of glutathione peroxidase decreased by the treatment of glutamate by 63.2 ± 3.1, 69.7 ± 4.8, and 84.1 ± 3.5%, respectively (Figure 4(B)). In the case of glutathione reductase, there was a similar tendency. In the glutamate-treated group, glutathione reductase was decreased by 53.2 ± 2.7% compared to the control group. Pretreatment of 1 μg/ml, 10 μg/ml and 100 μg/ml of FAE increased activity of glutathione reductase decreased by the treatment of glutamate by 58.1 ± 2.1, 75.3 ± 3.6, and 87.8 ± 5.4%, respectively (Figure 4(C)). In summary, these results suggested that FAE protect neuronal cell death against glutamate-induced oxidative stress through an antioxidant mechanism.

## Discussion

Many researchers have made many attempts to determine the cause of the neurodegenerative disorders, but the exact pathogenesis is still unknown and very complicated. Oxidative stress in brain is one of the pharmacological targets for the treatment of Alzheimer’s disease (McCully, 2009). The present study investigated the effect of fermented aloe (*Aloe arborescens*) extract on glutamate-induced neuronal excitotoxicity in HT22 cells and its mechanism of action. Furthermore, we investigated the effect of fermentation process of aloe extract on neuroprotective activity by comparison of aloe extract and fermented aloe extract. In general, the chemical profile was changed by the fermentation process and this change of chemical profile might affect the biological activity (Ryu and Kwon, 2012).

HT22 cell is a mouse hippocampal cell that is generally used to research glutamate oxidative stress. Glutamate triggers oxidative neuronal cell death in HT22 cells through necrosis and apoptosis. Although there are some potential reasons to explain the start and progress of Alzheimer’s disease, one of them is related to oxidative stress insulted by excitotoxicity. (Tan et al., 1998). Neurotoxicity of glutamate induced a decrease of cystine uptake through the glutamate/cystine antiporter and finally contributed to the reduction of glutathione production. (Pfeiffer et al., 2014). A small amount of glutathione prevents the glutathione redox cycle from working properly and it can inhibit the activity of glutathione redox cycle-related enzymes such as glutathione peroxidase (GPx) and glutathione reductase. As a result, free radicals and ROS were not properly removed in the cells, and this resulted in neuronal cell death by oxidative stress (Hong et al., 2020). The oxidative stress goes through downstream phases such as the increase of Ca^2+^ concentration, attenuation of mitochondrial membrane potential, depression of mitochondria, and finally to cell death (Andersen, 2004).

**Figure 5.**
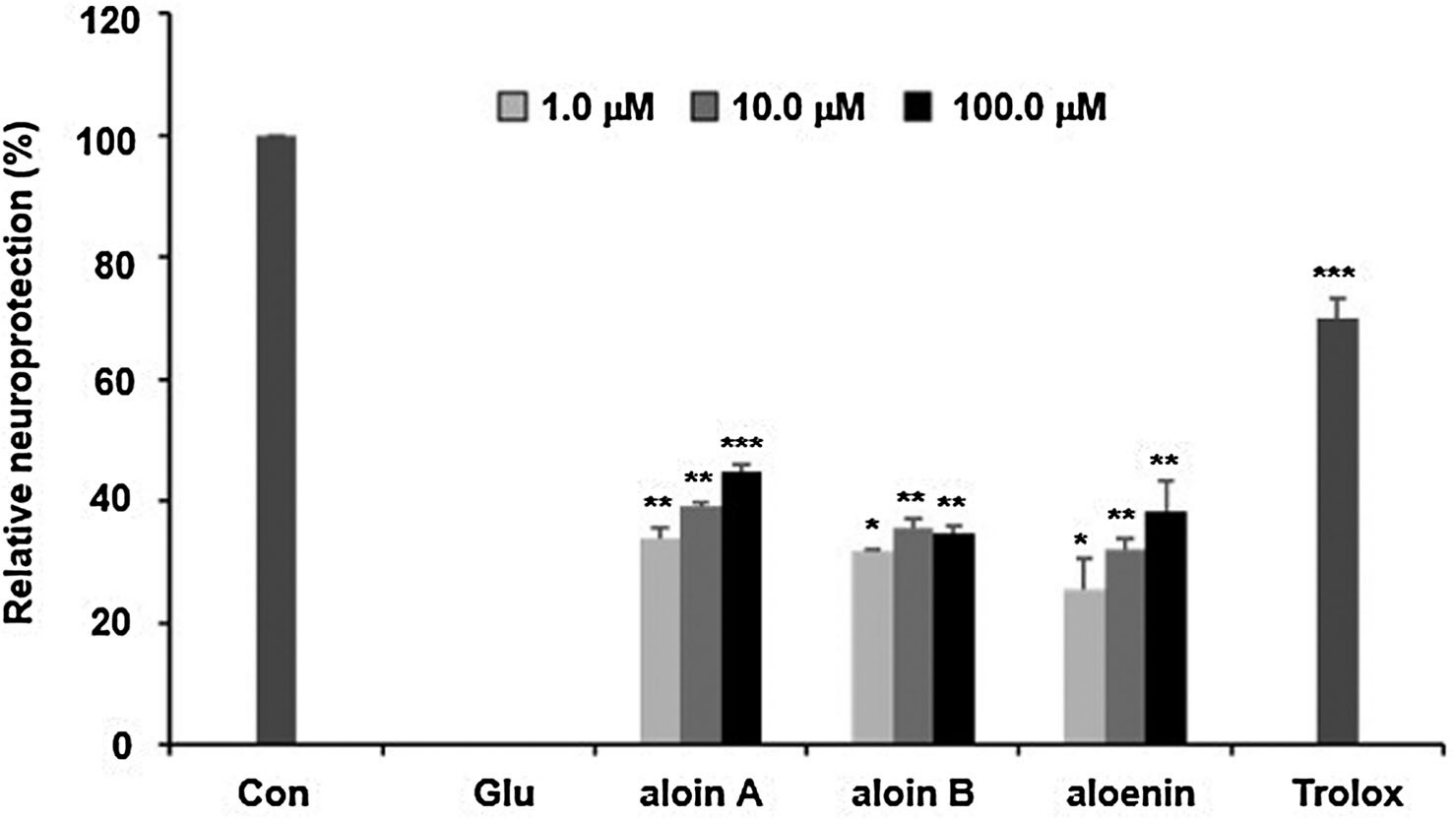
Neuroprotective activities of aloin A, aloin B and aloenin against glutamate-induced neuronal death of HT22 cells. Data are means ± S.D. *p < 0.05, **p < 0.01, and ***p < 0.001 versus the glutamate-treated group.

**Figure 6.**
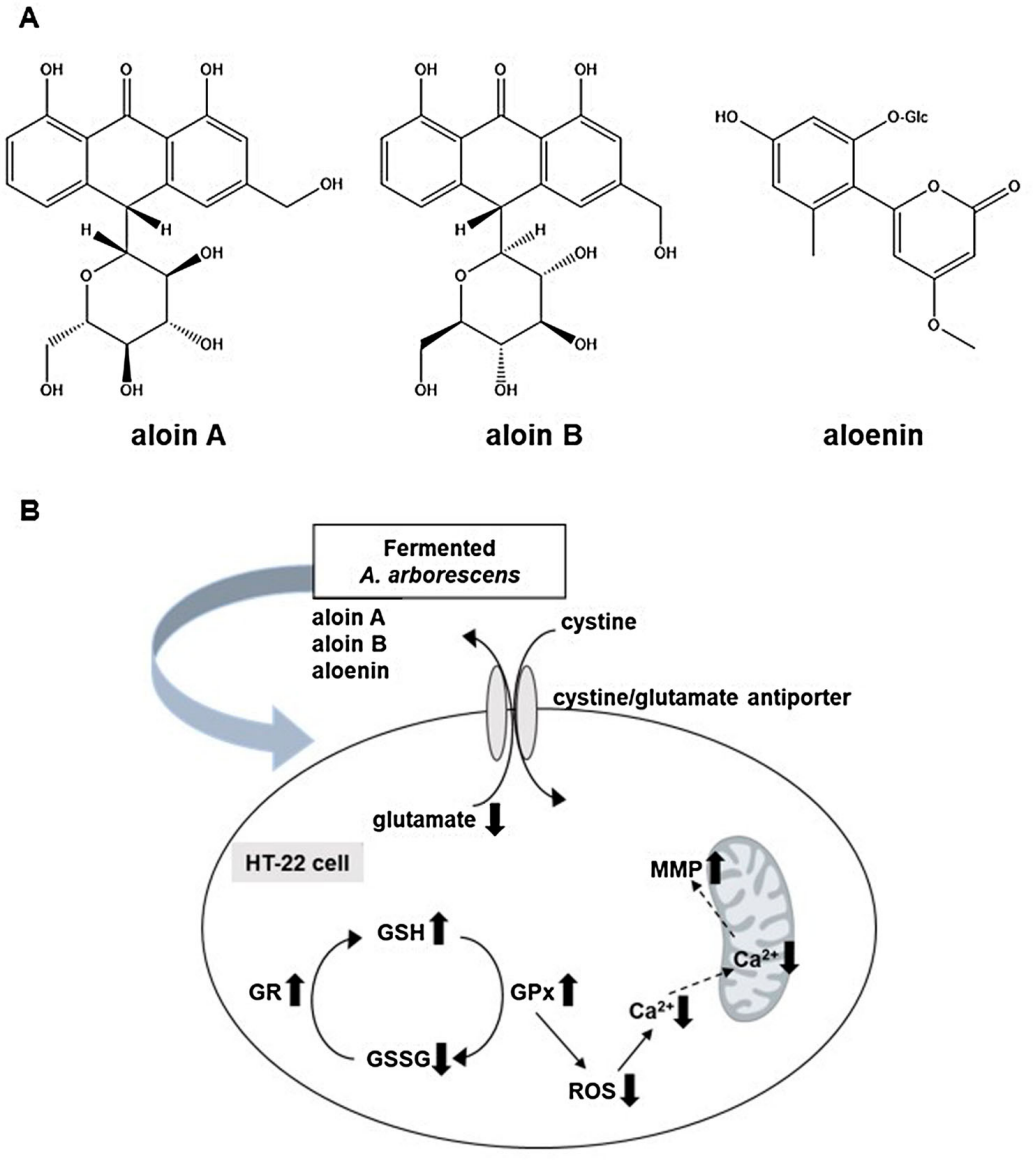
(A) Chemical structures of three active compounds of *A. arborescens.* (B) Schematic diagram of neuroprtection mechanism of fermented *A. arborescens* extract (FAE).

The above-mentioned mechanisms suggest that antioxidant activity is an important pharmacological target for developing the effective new medicine to treat Alzheimer’s disease. We firstly found the *A. arborescens* extract significantly improved the cell viability against glutamate-insulting neurotoxicity in HT22 cells in a dose-dependent manner. Furthermore, we found that the fermented *A. arborescens* extract with *Lactobacillus brevis* exerted more potent neuroprotective activity than *A. arborescens* extract. Many studies have shown that fermentation using probiotics such as *Lactobacillus* spp., *Bifidobacterium* spp., and *Saccharomyces* spp., changes the phytochemical composition and improves the pharmacological activity of some plant extracts (Trinh et al., 2007; Marazza et al., 2009; Dai et al., 2013). In this study, three bioactive compounds, aloin A, aloin B and aloenin were identified in the AE and FAE by HPLC-DAD analysis (Figure 6(A)). The contents of aloin A, aloin B and aloenin in the FAE were 48.42 ± 0.34, 24.98 ± 0.27, 8.22 ± 0.65 μg/mg, respectively. However, the contents of aloin A, aloin B and aloenin in the AE were 31.29 ± 0.62, 19.16 ± 0.32, 7.14 ± 0.27 μg/mg, respectively (Supplementary Data1A and B). This phytochemical composition difference was thought to have had a beneficial effect on the neuroprotective activity of FAE. In our assay system, aloin A, aloin B and aloenin actually had neuroprotective activity against glutamate-induced oxidative stress in HT22 cells (Figure 5). There is much evidence that fermentation of natural products using *Lactobacillus* related probiotics has contributed to an increase in bioactivity, especially antioxidant activity. (Ng et al., 2011; Oh et al., 2012; Shim et al., 2013). Our previous study showed enhancement of the neuroprotective activity of Gumiganghwal-tang fermented with *Lactobacillus* spp. against glutamate-induced neurotoxicity in HT22 cells (Weon et al., 2016). Our results showed that FAE protected HT22 cells against glutamate-insulting cell death by inhibiting ROS generation and Ca^2+^ influx. FAE also restored total glutathione amount, glutathione peroxidase, and glutathione reductase activity and recovered the mitochondrial membrane potential. In this process, increase of cystine uptake into the cells through the cystine/glutamate antiporter on the HT-22 cell membrane is expected to increase the production of glutathione to protected cell from oxidative stress (Figure 6(B)). Therefore, the neuroprotective effect of FAE against glutamate-induced cell death was mainly related to its antioxidative activity through attenuation of oxidative stress.

## Conclusion

In conclusion, we have shown that FAE could ameliorate glutamate-induced neurotoxicity in HT22 cells as assessed by MTT assay and that this may be attributed to the antioxidant activity caused by FAE. The present study suggests that FAE may be a potential therapeutic agent for the treatment of Alzheimer’s disease. However, further study is required to confirm the cognitive enhancing activity using animal behavioral test and its mechanisms of action by FAE.
